# FERMT1 contributes to the migration and invasion of nasopharyngeal carcinoma through epithelial–mesenchymal transition and cell cycle arrest

**DOI:** 10.1186/s12935-022-02494-1

**Published:** 2022-02-10

**Authors:** Lingling Li, Piao Li, Wei Zhang, Haiting Zhou, Ergang Guo, Guoqing Hu, Linli Zhang

**Affiliations:** 1 Department of Oncology, Tongji Hospital, Tongji Medical College of Huazhong University of Science and Technology, 1095 Jie Fang Avenue, Wuhan, 430030 Hankou China; 2 Department of Oncology, Jingzhou Central Hospital, The Second Clinical Medical College, Yangtze University, Jingzhou, China

**Keywords:** FERMT1, Nasopharyngeal carcinoma, Epithelial–mesenchymal transition, Cell cycle arrest

## Abstract

**Background:**

Fermitin family member 1 (FERMT1) is significantly overexpressed in human cancers and associated with poor prognosis, but its contributions to tumorigenesis and nasopharyngeal carcinoma (NPC) progression remain unclear.

**Methods:**

The public GEO database was examined to investigate the role of FERMT1. Immunohistochemistry (IHC) staining of FERMT1 was performed in NPC tissues to corroborate the results. Western blotting and qRT-PCR were performed to test the expression of related proteins and mRNAs. Cell counting kit-8 assay (CCK8 assay) and colony formation assays were carried out to investigate the association of FERMT1 expression with NPC cell proliferation. The wound healing assay and Transwell assay were used to detect the migration and invasion of NPC cells. Flow cytometric analysis was conducted to detect the cell cycle transition of NPC cells. Co-immunoprecipitation (Co-IP) was employed to identify the correlation of FEMRT1 and Nod-like receptor family protein 3 (NLRP3). Xenograft tumors were generated to investigate the effect of FERMT1 on the growth of NPC cells in vivo.

**Results:**

Here, we found that FERMT1 was upregulated in NPC tissues and correlated with the clinicopathological characteristics of NPC patients. Moreover, knockdown of FERMT1 significantly decreased cell proliferation, migration and invasion by mediating epithelial–mesenchymal transition (EMT) and cell cycle arrest of NPC cells both in vitro and in vivo. Knockdown FERMT1 inhibited EMT through directly binding to the NLRP3 and inhibited NF-kB signaling pathway.

**Conclusion:**

These data indicated that FERMT1 could be a good potential therapeutic target for NPC treatment.

**Supplementary Information:**

The online version contains supplementary material available at 10.1186/s12935-022-02494-1.

## Background

Nasopharyngeal carcinoma (NPC) is a malignant epithelial carcinoma originating from the nasopharynx and has a crucial correlation with the Epstein–Barr virus [1]. The high incidence area of this tumor is mainly in southern China and Southeast Asia [2]. As multimodality therapy progresses, such as intensity-modulated radiation therapy, chemotherapy, targeted therapy, and immunotherapy, the effective rate of treatment is improved. However, there are still patients who fail treatment, and the main reason for treatment failure is distant metastasis. As far as we known, NPC metastasis involves a complex process including various factors and cellular signaling pathways. However, the specific mechanism of NPC metastasis is unclear. Accordingly, it is essential to explore the underlying molecular mechanisms of NPC metastasis.

Fermitin family member 1 (FERMT1) is expressed in most epithelial tissues. FERMT1 primarily participates in different cellular processes, such as cell adhesion, cell motility and cell migration. Defects in FERMT1 cause Kindler syndrome (KS), a genetic disease that is characterized by fragile skin and an increased risk of squamous cell carcinoma [3]. Furthermore, growing evidence has confirmed that FERMT1 might play a role in tumor proliferation, metastasis, apoptosis, and tumor angiogenesis [4]. Liu et al. showed that FERMT1 was strongly upregulated in colon cancer and could activate the transcriptional activity of β-catenin to drive epithelial–mesenchymal transition (EMT) [5]. Meanwhile, more evidence has suggested that FERMT1 has a role in many types of tumors, such as skin cancer, lung cancer, bladder cancer, and pancreatic cancer [6, 7]. However, few studies have evaluated the relationship between FERMT1 and NPC.

Increasing evidence indicates that EMT plays a crucial role in the onset and development of NPC [8–10]. EMT refers to a process in which epithelial cells lose their epithelial phenotype and obtain characteristic mesenchymal features. During the process of EMT, epithelial cells lose their junction and top basal polarity, recombining their cytoskeleton, changing the signal program that defines cell shape, and recoding gene expression [11]. Indeed, this increases the motility of individual cells and leads to the development of an aggressive phenotype, which may eventually cause these cells to separate from the primary tumor and migrate to other tissues [12]. In addition, EMT is characterized by loss of the epithelial cell adhesion molecule E-cadherin and increased expression of mesenchyme markers such as N-cadherin, vimentin, snail, and twist [13]. In addition, ZEB1 induces EMT by recruiting SMARCA4/BRG1 to inhibit the E-cadherin promoter [14]. At the same time, normal cell growth and metabolism are precisely controlled by the cell cycle. Protein kinase complexes composed of cyclin and cyclin-dependent kinase (CDK) play a crucial role in identifying the sequential development of cells [15]. From the molecular point of view, cyclin is the regulatory subunit of the activated heterodimer. At the same time, CDKs are the catalytic subunit that coordinates entry into the S phase of the cell cycle [16]. Various cyclin-CDK complexes precisely identify downstream targeted proteins and promote the expression of cyclins and enzymes related to DNA replication [17]. Disorder of cell cycle components can cause uncontrollable tumor cell proliferation and cancer.

Nod-like receptor family protein 3 (NLRP3) belongs to a typical inflammasome family of leucine-rich repeat containing proteins. It can mediate cancer pathogenesis by mediating apoptosis proteins and immune responses [18]. Yin et al. found that NLRP3 improved glioma cell viability, metastasis and EMT and inhibited cell apoptosis [19]. In previous studies, it was well known that NF-kB acted as the upstream of NLRP3 [20]. Furthermore, the activation of NF-kB was found to activate the expression of potent EMT inducers, such as snail and zeb [21]. However, there were no studies reported the relation between FERMT1 and NLRP3. The aim of our study was to investigate the effects of FERMT1 on NPC cell viability through regulating NF-kB/NLRP3 signaling pathway.

In this study, our results showed that compared with normal tissue, the expression of FERMT1 was upregulated in NPC, and its overexpression indicated a poor prognosis in patients with NPC. In vitro and in vivo, knockdown of FERMT1 suppressed EMT and induced cell cycle arrest to inhibit NPC proliferation, migration and invasion. Our study reported that FERMT1 mediated EMT through binding to NLRP3. Our findings suggested that FERMT1 might be a potential therapeutic target for NPC treatment.

## Methods

### Tissue samples

From January 2015 to August 2018, ten samples of noncancerous inflammatory nasopharyngeal epithelial tissues and 109 primary NPC tumor tissues were obtained from the Tongji Hospital of Tongji Medical College, Huazhong University of Science and Technology. All tissues were collected at the first diagnosis. We divided the patients into high and low groups by taking the median score as the cut-off value. Two independent histopathologists identified the diagnosis of NPC in each patient. This study attained approval from the Research Ethics Committee of the Tongji Hospital of Tongji Medical College, Huazhong University of Science and Technology.

### Immunohistochemistry

Immunohistochemistry (IHC) staining and scoring were performed as previously mentioned. The sections were incubated with FERMT1 antibody (22215-1-AP, 1:200 dilution, Proteintech). Regarding FERMT1 expression, these sections were then subdivided into two categories: 0 and 1+ were defined as low expression, and 2+ and 3+ were defined as high expression. In addition, tissue IHC was used to detect FERMT1, E-cadherin, N-cadherin, vimentin, CDK4, CDK6, cyclinD1 and cyclinB1 expression with the indicated antibodies for xenograft mouse tissues, including anti-FERMT1 (22215-1-AP, Proteintech, China), anti-E-cadherin (14472, Cell Signaling Technology, USA), anti-N-cadherin (22215-1-AP, Proteintech, China), anti-vimentin (22215-1-AP, Proteintech, China), anti-CDK4 (22215-1-AP, Proteintech, China), anti-CDK6 (22215-1-AP, Proteintech, China), anti-cyclinB1 (22215-1-AP, Proteintech, China), and anti-cyclinD1 (22215-1-AP, Proteintech, China).

### Cell culture

Human NPC cell lines (CNE2, HK1, CNE1, HNE1, HONE1) and nasopharyngeal immortal epithelial cells (NP69) were acquired from the Cancer Research Institute of Central South University (Changsha, China). CNE2, HONE1 and HNE1 are epithelial cell lines of poorly differentiated nasopharyngeal squamous cell carcinoma. CNE1 is a well-differentiated squamous cell carcinoma, and HK1 is a well-differentiated, EBV-positive squamous cell carcinoma. CNE2 cell lines were identified by STR DNA profiling analysis. All NPC cell lines were routinely tested for mycoplasma contamination and cultured in RIPM 1640 medium (Promoter Biotech, China) supplemented with 10% fetal bovine serum (FBS, Gibco, South America), while NP69 cells were cultured with keratinocyte-SFM medium with 0.2 μg/ml EGF (Gibco, USA) and 30 μg/ml bovine pituitary extract (Gibco, USA). All cells were cultured in a humidified atmosphere of 37 °C and 5% CO_2_.

### Construction of the lentivirus vectors and cell transfection

The lentiviral vector was constructed and used for cell transduction as described previously to establish stably transfected cell lines for subsequent experimentation. FERMT1 shRNA lentiviral particles were obtained from GeneChem (GV112, Shanghai, China). CNE2 and HK1 cells were transfected with FERMT1 shRNA lentiviral particles according to the manufacturer’s instructions. Puromycin (3 µg/ml, Sigma, USA) was used to select stable cell lines in selective medium for at least 2 weeks. Western blotting and real-time PCR were used to detect the efficiency of transfection. The target sequences of FERMT1 shRNA were as follows:

shRNA 1: CAGCTTCAGGTTCATCAGTAA;

shRNA 2: GAGCAGCTGCTCTTACGATTT;

shRNA 3: CAGCTCTACAGTACCACATTA.

### Western blotting

To evaluate the protein expression, we performed western blotting. Total protein extracts were prepared with radioimmunoprecipitation assay (RIPA) buffer (P0013K, Beyotime Biotechnology, China). The proteins were measured in a microplate reader (Synergy H1, Biotek, USA) by Beyotime protein assay reagent using a wavelength of 562 nm. The proteins were separated using sodium-dodecyl-sulfate polyacrylamide gel electrophoresis (SDS–PAGE) and then transferred onto polyvinylidene fluoride (PVDF) membranes (Millipore, Billerica, MA, USA). Blots were incubated with the primary antibodies at 4 °C overnight. The next day, the blots were incubated with secondary antibodies for 1 h after washing and visualized with West Dura extended duration substrate (34580, Thermo Fisher Scientific, USA). Anti-FERMT1 (22215), anti-CDK4 (11026), anti-CDK6 (14052), anti-cyclinD1 (60186), anti-N-cadherin (22018), anti-vimentin (10366), anti-cyclinB1 (55004), anti-ZEB1 (21544) and anti-GAPDH (60004) were purchased from Proteintech (Wuhan, China). Anti-E-cadherin (#14472) and anti-phospho-Rb (#9301) were obtained from Cell Signaling Technology (USA). Anti-snail (ab117866) and anti-twist1 (ab50887) antibodies were purchased from Abcam (USA). Anti-NLRP3 (M035175F), anti-total-NF-kB (T55034F), anti-Phosphorylated-NF-kB (p-NF-kB) (TP56371F), anti-total-IkBα (T55026F) and anti-Phosphorylated-IkBα (p-IkBα) (TP56280F) were obtained from Abmart (Shanghai, China).

### Reverse transcription and quantitative real-time polymerase chain reaction (qRT-PCR)

According to the manufacturer’s instructions, total RNA was prepared using TRIzol reagent (Takara, Dalian, China). cDNA synthesis was carried out using HiScript II Q RT SuperMix for qPCR (Vazyme Biotech, Nanjing, China). Real-time PCR was performed with a fast real-time PCR system (7900HT, Applied Biosystems, USA). qPCR was used to detect the mRNA expression level using ChamQ Universal SYBR qPCR Master Mix (Vazyme Biotech, Nanjing, China). The sequences of the primers were shown in Additional file 1: Table S1.

### Cell counting kit‐8 (CCK8) assay

First, NPC cells were implanted at 800 cells per well into 96-well plates. For the CCK-8 assay (MCE, HY-K0301), CCK-8 reagent (10% of the serum-free medium volume) was added to the cells and incubated for another 1 h at 37 °C. The absorbance was determined by a microplate reader (BioTek, Winooski, VT, USA) at 450 nm to assess the cell proliferation ability. Cell viability was measured daily for six consecutive days.

### Colony formation assay

The growth of NPC cells was evaluated with a colony formation assay. NPC cells were uniformly implanted at 200 per well in 6-well plates and incubated at 37 ℃ for 10–14 days. More than 50 cells under the microscope were regarded as valid clones. Then, the colonies were fixed with methanol for 20 min and stained with 0.1% crystal violet for 20 min. Three replicates were set for each group. The colonies were photographed and counted by three researchers independently. GraphPad Prism 8 software was used to analyze all data.

### Wound-healing experiment

The migration ability of NPC cells was detected with a wound-healing experiment. The cells were plated in 24-well culture plates with RPMI 1640 medium supplemented with 10% FBS and were cultured at 37 °C until cell monolayer confluence. A straight line was drawn at the bottom of the 24-well plate with a 20-µL pipette tip, and then the cells were grown at 37 °C in serum-free RPMI 1640 medium. The migration of the cells was captured under a microscope at 0, 12, and 24 h after scratching. The scratch distances were determined by ImageJ analysis software (National Institutes of Health, USA). Each experiment was performed at least three times.

### Transwell migration and invasion assay

NPC cell migration and invasion were determined by Transwell migration and invasion assays. Cell suspensions with 200 µL serum-free RPMI 1640 medium were implanted 8-μm-pore with or without Matrigel-coated Transwell chambers (Corning Costar, Cambridge, MA) to a density of 1 × 10^5^ cells/well, and then the inserts were held in the lower chamber with 500 μL of RPMI 1640 medium supplemented with 10% FBS and incubated at 37 °C. After 36 h, cells that were on the inside of the Transwell inserts were wiped with a cotton swab. Then, cells that had migrated to the lower surface of the membrane were fixed and stained with crystal violet. Five random fields were photographed, and the cells were counted to calculate the average number of migrated cells. Each experiment was performed at least three times.

### Cell cycle analysis

The cell cycle distribution was assessed by the cell cycle detection kit (Promoter, Wuhan, China) of NPC cells after silencing FERMT1. Cells were digested, collected, and fixed overnight using ice-cold 75% ethanol at − 20 °C. According to the manufacturer’s instructions, the cells were treated with RNaseA at 37 °C for 30 min and stained with propidium iodide (PI) at 37 °C in the dark for 30 min. The samples were detected by a FACSCanto II flow cytometer (BD Biosciences, USA).

### Co-immunoprecipitation

Co-immunoprecipitation (co-IP) assays were performed as previously described. NPC cells were harvested and lysed with cold co-immunoprecipitation buffer (Promoter, Wuhan, China). The supernatants were subjected to immunoprecipitation with anti-FERMT1 antibody or anti-NLRP3 antibody conjugated with protein A/G magnetic beads (MCE, HY-K0202). Bound protein was detected by the western blotting analysis mentioned above.

### In vivo xenograft assay

The results were further validated using a xenograft mouse model. Four- to five-week-old female BALB/c nude mice (GemPharmatech, Nanjing, China) were reared in specific pathogen-free (SPF) conditions, constant temperature and constant humidity, and sufficient food and water every day. According to the Animal Study Guidelines of Tongji Hospital, Tongji Medical College, Huazhong University of Science and Technology Animal Care Facility and National Institutes of Health guidelines, all animal experiments were strictly raised. The mice were randomized into two groups, and each group contained eight mice: named HK1 and HK1-shFERMT1 groups. NPC cells (2.0 × 10^6^/100 µL) were implanted subcutaneously into the right axilla of each mouse. The size of the tumor was measured using a caliper every 3 days. The tumor volume calculation formula was as follows: tumor volume = (long length × short length^2^)/2. The experiment was terminated after 27 days of tumor growth. The anesthetized mice were euthanized by cervical dislocation. This study was reviewed and approved by the Ethics Committee of Tongji Hospital, Tongji Medical College, Huazhong University of Science and Technology.

### The collection and analysis of data

We downloaded the expression profile of genes from the Gene Expression Omnibus (GEO) (https://www.ncbi.nlm.nih.gov/geo/), and the download data format was MINIML. GSE12452 was composed of mRNA of laser-captured epithelium from 31 NPC and 10 non-NPC nasopharynx tissues from the Taiwanese case–control cohort. mRNA expression was analyzed with Affymetrix Human Genome U133 Plus 2.0 Array. Quantile normalization of microarray data was used for the log2-transformed intensity values as a method for between array normalization to obtain a similar intensity distribution across arrays. The Cancer Genome Atlas (TCGA) databases (https://www.cancer.gov/tcga/) was used and downloaded to analyze the FERMT1 in the head and neck squamous cell carcinoma (HNSCC).

### Gene set enrichment analysis

According to the expression of FERMT1, gene set enrichment analysis (GSEA) was performed to identify the significantly different genes. GSEA software (UC San Diego and Broad Institute, San Diego, CA, USA) was used to analyze the GSEA of hallmarks. Gene set permutations were used 1000 times per analysis. The normalized enrichment score (NES), nominal P value, and false discovery rate (FDR) q-value indicated the significance of the enrichment results.

### RNA sequencing analysis

After lentivirus FERMT1-shRNA was transfected into HK1 cells, the total RNA of each sample was extracted. The RNA concentration and purity were determined with a NanoDrop2000 (Thermo Fisher, USA). RNA sequencing (RNA-Seq) analysis was conducted by GeneChem (Shanghai, China). Differential expression gene (DEG) analysis was performed using DESeq2 and EdgeR software. Finally, padj value < 0.05 and |log_2_foldchange|> 2 were regarded as the thresholds for defining differential expression.

### Statistical analysis

SPSS software (version 26.0; SPSS, Chicago, IL, USA) was used for statistical analysis. Means ± standard deviation (SD) were used to express the data. The chi-square test or Fisher’s exact test was applied to analyze the relationship between FERMT1 expression and clinicopathological characteristics. Kaplan–Meier analysis and a log-rank test were used for survival comparison. Statistical significance was determined with Student’s t test or two-way analysis of variance (ANOVA). Differences were considered statistically significant at a P value < 0.05 (*P < 0.05, **P < 0.01, ***P < 0.001).

## Results

### FERMT1 is highly expressed in NPC tissues and cells

**Fig. 1:**
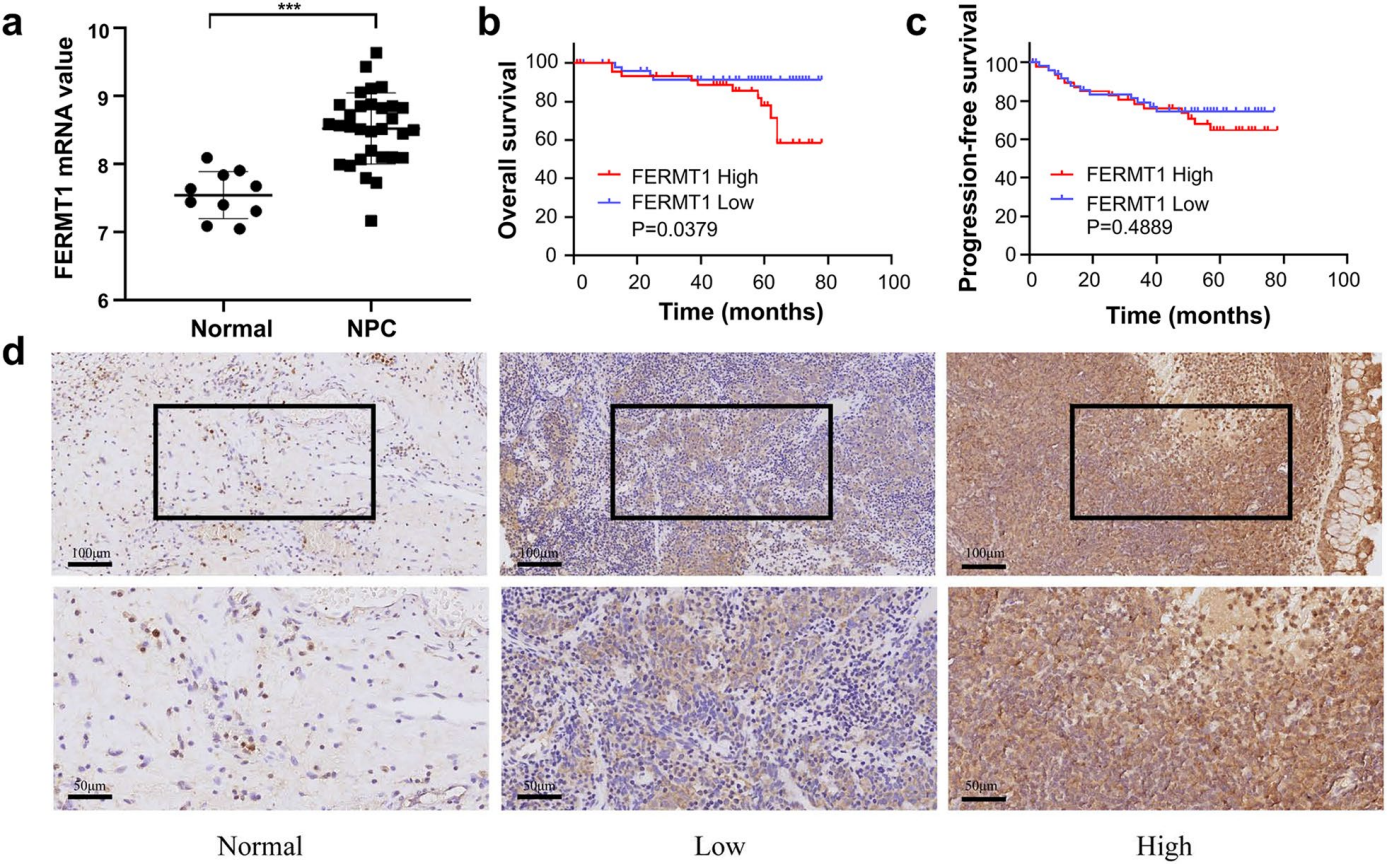
FERMT1 expression is upregulated and associated with poor prognosis in NPC patients. **a** FERMT1 expression was analyzed in public GEO databases (GEO: GSE12452). **b** Kaplan–Meier overall survival curves for NPC patients stratified by high versus low FERMT1 expression. **c** Kaplan–Meier progression-free survival curves for NPC patients stratified by high versus low FERMT1 expression. **d** Representative images of IHC staining showing negative, low, and high FERMT1 expression in NPC tissues; upper, original magnification, ×200; lower, original magnification, ×400

To investigate the role of FERMT1 in NPC, we examined public GEO databases (GEO: GSE12452). The results demonstrated that FERMT1 expression was elevated in NPC tissues compared to paired normal tissues (Fig. 1a). We performed immunohistochemistry (IHC) staining of FERMT1 in NPC tissues to corroborate the above results. Both the cytoplasm and nucleus of NPC cells displayed FERMT1 expression (Fig. 1d). High expression of FERMT1 was observed in 54/109 (49.5%) NPC samples (Table 1). We found that tumor tissues showed a relatively high level of FERMT1 compared to noncancerous tissues (P = 0.016, Table 1). However, high FERMT1 expression was not statistically correlated with age, sex, N classification, or histologic subtype, but it was significantly correlated with T classification (P = 0.001), M classification (P = 0.035), and clinical stage (P = 0.001). In addition, Kaplan–Meier analysis showed that the expression of FERMT1 in patients with NPC was significantly correlated with the overall survival rate. As shown in Fig. 1b, the overall survival of NPC patients with high FERMT1 expression was shorter than that of NPC patients with low FERMT1 expression (P = 0.0379), suggesting that high expression of FERMT1 indicated a poor prognosis for NPC patients. Nevertheless, the expression of FERMT1 was not associated with the progression-free survival of patients with NPC (Fig. 1c). Univariate analysis showed that N classification, M classification and clinical stage were independent factors for the OS rates of patients with NPC (P = 0.005, P = 0.03, and P = 0.012, respectively). Nevertheless, the results were not verified in the multivariate analysis (Additional file 1: Table S2).

**Table 1:** Association between FERMT1 expression and clinicopathological characteristics in NPC patients

Characteristics	Total	High expression FERMT1	P value
Number, n (%)			–
Tumor tissues	109	54(49.5)	0.016
Non	10	1	
Age (years) (n[%])			
< 60	92	46	0.824
≥ 60	17	8	
Gender (n [%])			
Female	33	12	0.07
Male	76	42	
T classification			0.001
T1–2	42	12	
T1	14	3	
T2	28	9	
T3–4	67	42	
T3	35	24	
T4	32	18	
N classification			
N0–1	39	16	0.184
N0	12	3	
N1	27	13	
N2–3	70	38	
M classification			0.035
M0	96	44	
M1	13	10	
Clinical stage			0.001
I–II	20	3	
III–IV	89	51	
Histologic subtypes			0.403
WHO type I	1	1	
WHO type II	11	4	
WHO type III	97	49	

Keratinizing squamous cell carcinoma

Nonkeratinizing differentiated carcinoma

Nonkeratinizing undifferentiated carcinoma

P < 0.05

**Fig. 2:**
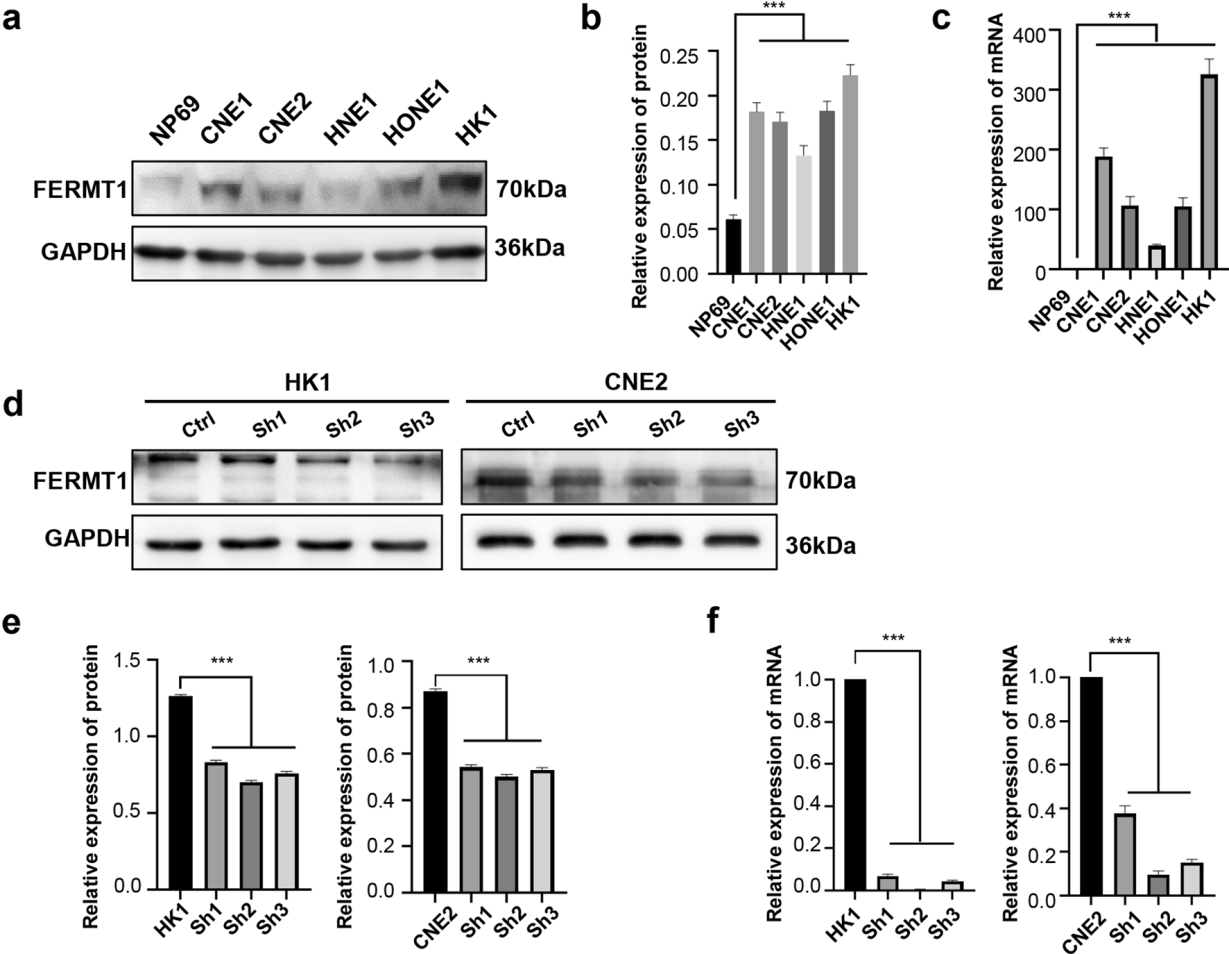
High expression of FERMT1 in NPC cells. **a**, **b** Western blotting analysis was applied to detect the protein expression level of FERMT1 in NP69 and different NPC cells. **c** qRT-PCR analysis was used to detect the mRNA expression level of FERMT1 in NP69 and different NPC cells. **d**, **e** Western blotting analysis was used to detect the interference effects of shFERMT1 on HK1 and CNE2 cells. **f** qRT-PCR analysis was used to detect the interference effects of shFERMT1 on HK1 and CNE2 cells; *P < 0.05, **P < 0.01, ***P < 0.001

To further verify the significance of FERMT1 in NPC, we assessed the mRNA and protein levels of FERMT1 in five NPC cell lines (CNE1, CNE2, HNE1, HONE1, and HK1) and immortalized nasopharyngeal epithelial NP69 cells. Compared with NP69, FERMT1 protein and mRNA levels were upregulated in all NPC cells (Fig. 2a–c).

### FERMT1 knockdown suppresses NPC cell proliferation, migration and invasion in vitro

**Fig. 3:**
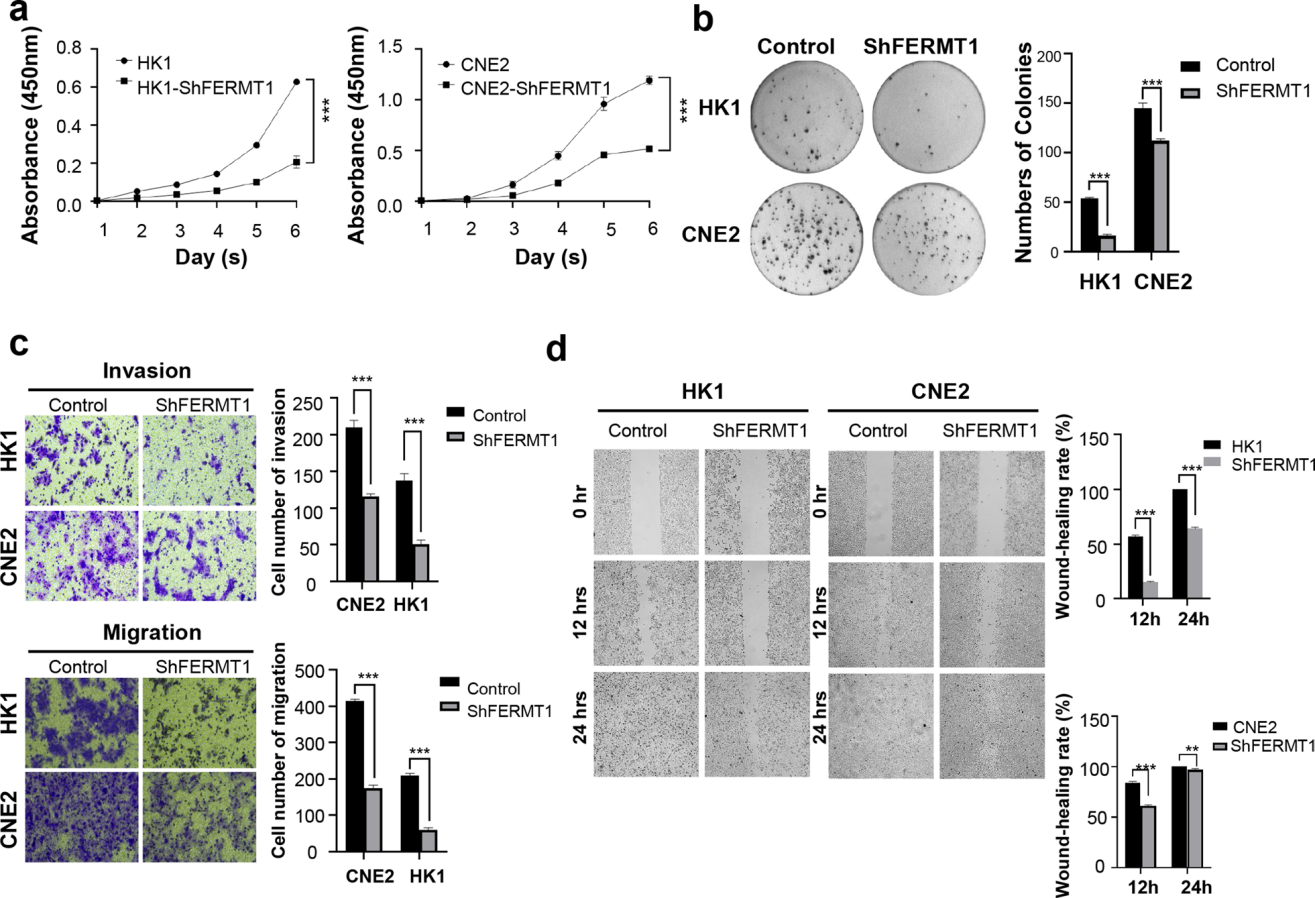
FERMT1 promotes the proliferation, invasion and migration of NPC cells. **a** The CCK-8 assay and **b** colony formation assays were used to detect the proliferation of HK1 and CNE2 cells. **c** The transwell invasion and migration assay were used to detect the invasion and migration ability of HK1 and CNE2 cells. **d** The wound healing assay was used to detect the migration ability of HK1 and CNE2 cells; *P < 0.05, **P < 0.01, ***P < 0.001

To confirm the function of FERMT1 in NPC, we first explored whether FERMT1 knockdown with lentiviral shRNAs had any effects on cell proliferation, invasion and migration. As shown in Fig. 2d–f, the mRNA and protein expression levels of FERMT1 sharply decreased after transfection with three shRNAs targeting different positions of FERMT1, among which FERMT1 shRNA3 had the greatest knockdown efficiency on FERMT1 expression in CNE2 and HK1 cells. Therefore, FERMT1 shRNA3 was used for further functional and mechanistic investigations, named HK1-shFERMT1 and CNE2-shFERMT1, respectively. Indeed, FERMT1 knockdown suppressed cell proliferation. Cell counting kit-8 assay (CCK8 assay) and colony formation assays were performed to investigate the association of FERMT1 expression with NPC cell proliferation. The CCK-8 assay showed that knockdown of FERMT1 notably attenuated the proliferation of both HK1 and CNE2 cells (Fig. 3a). The colony formation assay results confirmed that FERMT1 knockdown significantly resulted in a lower number of colonies than the control groups in NPC cells (Fig. 3b). In addition, FERMT1 knockdown significantly inhibited HK1 and CNE2 cell migration, as evidenced by the wound healing assay (Fig. 3d). Consistently, invasion and migration of HK1 and CNE2 cells detected were also decreased under FERMT1 knockdown by the Transwell assay (Fig. 3c). These results suggested that FERMT1 might exert important functions in promoting NPC growth and metastasis.

### FERMT1 knockdown restrains EMT in NPC cells

**Fig. 4:**
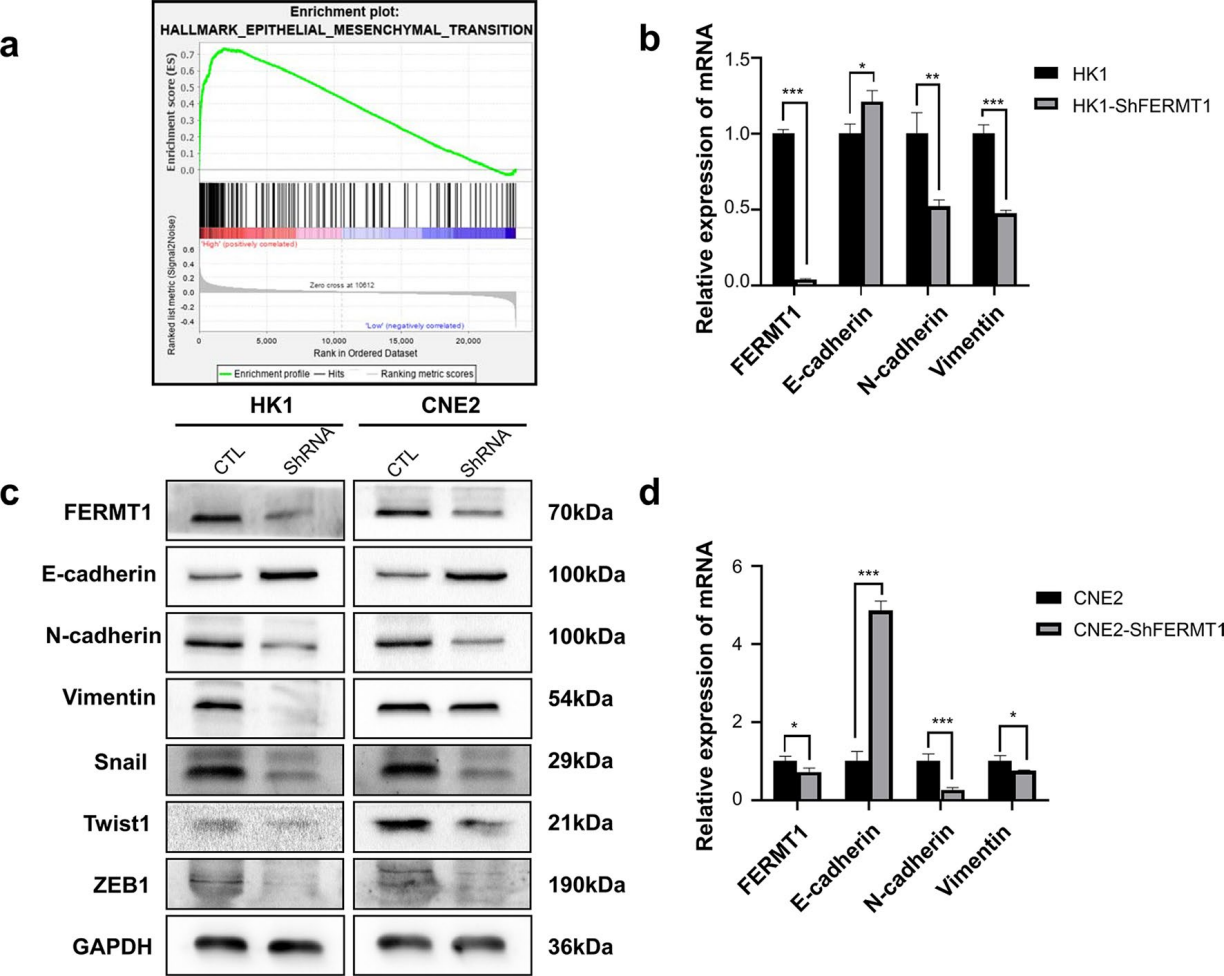
Knockdown of FERMT1 induces EMT in NPC cells. **a** GSEA suggested that FERMT1 was enriched in the hallmark of EMT. qRT-PCR was used to analyze EMT-related mRNA levels in **b** HK1 cells and **d** CNE2 cells. **c** Western blotting was applied to detect EMT-related protein levels in HK1 and CNE2 cells; *P < 0.05, **P < 0.01, ***P < 0.001

To evaluate the effects of FERMT1 on the phenotypes of NPC, we used published NPC datasets (GSE12452) to compare the gene profiles of NPC samples with high and low expression of FERMT1 through GSEA. We found that genes significantly correlated with FERMT1 were enriched in the hallmark of EMT (NES = 1.56, P = 0.01, FDR = 0.27, Fig. 4a), which suggested that FERMT1 might play a regulatory role in EMT. To confirm this hypothesis, we then detected EMT marker proteins to explore whether the effect of FERMT1 on cell migration and invasion was a result of EMT. Our results showed that the protein expression of E-cadherin was significantly elevated after FERMT1 knockdown in HK1 and CNE2 cells, whereas that of N-cadherin, vimentin, Snail, Twist1 and ZEB1 was significantly lower (Fig. 4c). The gene levels of EMT markers also showed the same results (Fig. 4b, d). These data indicated that FERMT1 might regulate NPC metastasis via the EMT pathway.

### FERMT1 knockdown induces G0/G1 cell cycle arrest in NPC cells

**Fig. 5:**
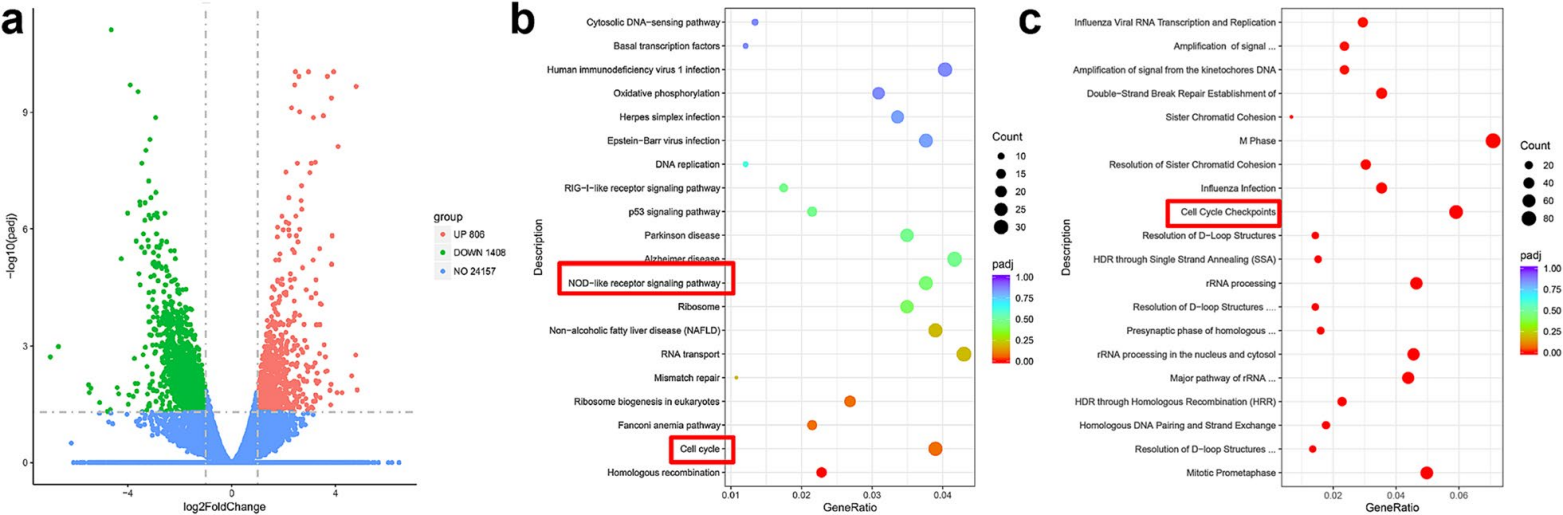
Downstream transcriptional genes of FERMT1 in NPC cells. **a** Volcano diagram depicting the 2214 differentially expressed genes (DEGs) from the RNA sequencing in that HK1 cells were transfected with shFERMT1. Red represents upregulated genes, green represents downregulated genes, and blue represents nonsignificant genes. **b** KEGG **c** REACTOME pathway analysis of the DEGs

**Fig. 6:**
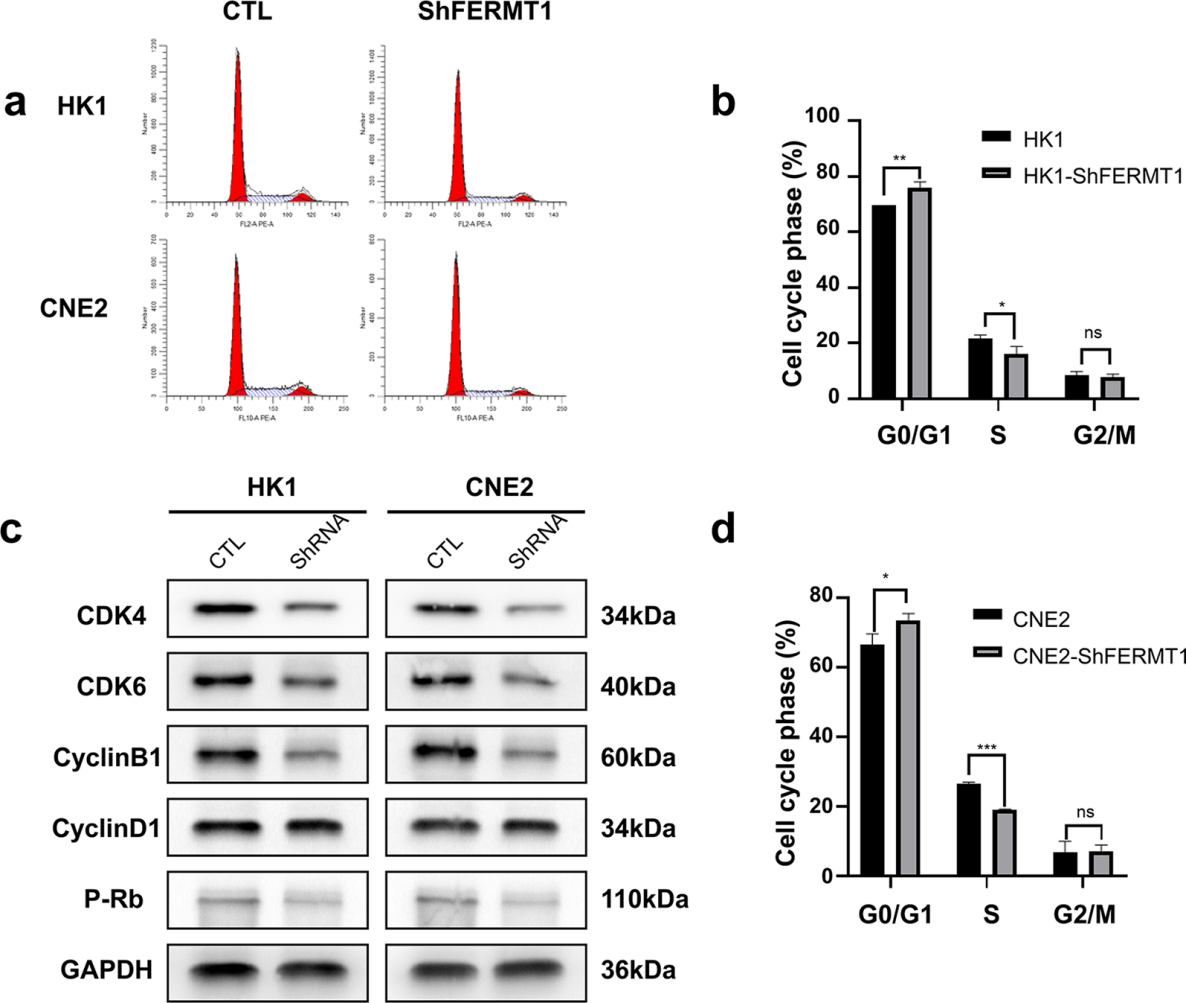
Silencing of FERMT1 induces G0/G1 cell cycle arrest in NPC cells. **a**, **b**, **d** The cell cycle was assessed by flow cytometric analysis in HK1 and CNE2 cells. **c** Western blotting analysis was used to analyze the indicated proteins in HK1 and CNE2 cells transfected with shFERMT1 lentiviral particles; *P < 0.05, **P < 0.01, ***P < 0.001

To further explore the underlying mechanism by which FERMT1 might play in the metastasis of NPC cells, we conducted RNA sequencing analysis in HK1 cells transfected with shRNA. At the same time, HK1 cells were included as a control group. As shown in Fig. 5a, a total of 2214 DEGs (806 upregulated genes and 1408 downregulated genes) were recognized between the shRNA-FERMT1 and negative control groups. In addition, KEGG and REACTOME pathway analyses were used to predict the DEG functional and signaling pathway enrichment. We identified that the DEGs were enriched in the following pathways: homologous recombination, cell cycle, NOD-like receptor signaling pathway, fanconi anemia pathway and ribosome biogenesis in eukaryotes in KEGG pathway analysis (Fig. 5b). The DEGs were significantly enriched in the M phase, cell cycle checkpoints and mitotic prometaphase for REACTOME pathway analysis (Fig. 5c). To better understand the mechanism of FERMT1 on growth-suppressive activity in NPC cells, we next analyzed whether FERMT1 regulated cell cycle progression. Flow cytometric analysis showed that FERMT1 knockdown inhibited the cell cycle transition of CNE2 and HK1 cells from G1 to S phase (Fig. 6a–b, d). The changes in important regulators of cell cycle progression were further demonstrated by western blotting. This study showed that FERMT1 knockdown markedly reduced the expression of CDK4, CDK6, cyclin B1 and pRb (Fig. 6c). Nonetheless, the expression of cyclin D1 did not change.

### FERMT1 knockdown inhibits the EMT of NPC cells via NF-kB/NLPR3 signaling

**Fig. 7:**
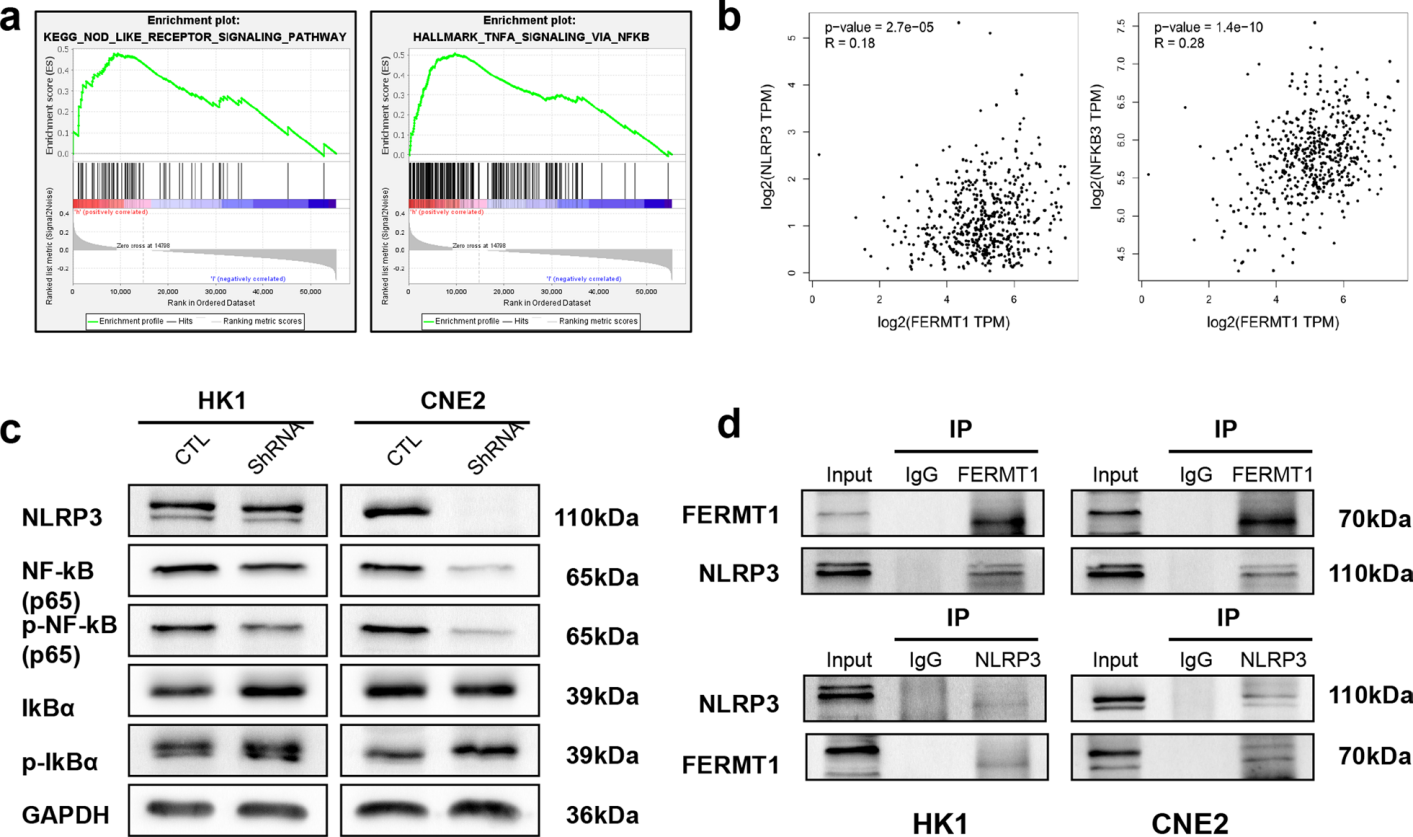
FERMT1 knockdown inhibits the EMT of NPC cells via NF-kB/NLPR3 signaling. **a** GSEA suggested that FERMT1 was enriched in the NOD like receptor signaling pathway and NF-kB signaling based on the TCGA dataset. **b** Pearson correlation analysis of FERMT1 and NLRP3 protein expression and Pearson correlation analysis of FERMT1 and NF-kB protein expression in the TCGA dataset. **c** Western blotting was applied to detect NF-kB/NLPR3 signaling pathway related protein levels in HK1 and CNE2 cells. **d** Co-IP detected FERMT1 and NLRP3 interaction in HK1 and CNE2 cells

We explored the underlying mechanism of FERMT1 in the EMT of NPC cells. Considering the limitation of small sample size in the GEO databases and the lack of specific NPC samples in TCGA databases, we used TCGA to analyze FERMT1 in the HNSCC. We found that genes significantly correlated with FERMT1 were enriched in the NOD like receptor signaling pathway (NES = 1.81, P = 0.02, FDR = 0.40) and NF-kB signaling (NES = 1.88, P = 0.03, FDR = 0.14) through GSEA analysis (Fig. 7a). This result was consistent with our RNA-seq data. At the same time, we found that FERMT1 and NLPR3 were positively correlated, and so were FERMT1 and NF-kB (Fig. 7b). Subsequently, to verify the pathway analysis results, we conducted the western blotting experiment. As shown in Fig. 7c, decrease of NLRP3 and p-NF-kB levels occurred due to FERMT1 knockdown in HK1 and CNE2 cells and the p-IkBα levels were increased. Co-IP revealed the association between FERMT1 and NLRP3 (Fig. 7d).

### FERMT1 knockdown restrains the growth of xenograft tumors in vivo

**Fig. 8:**
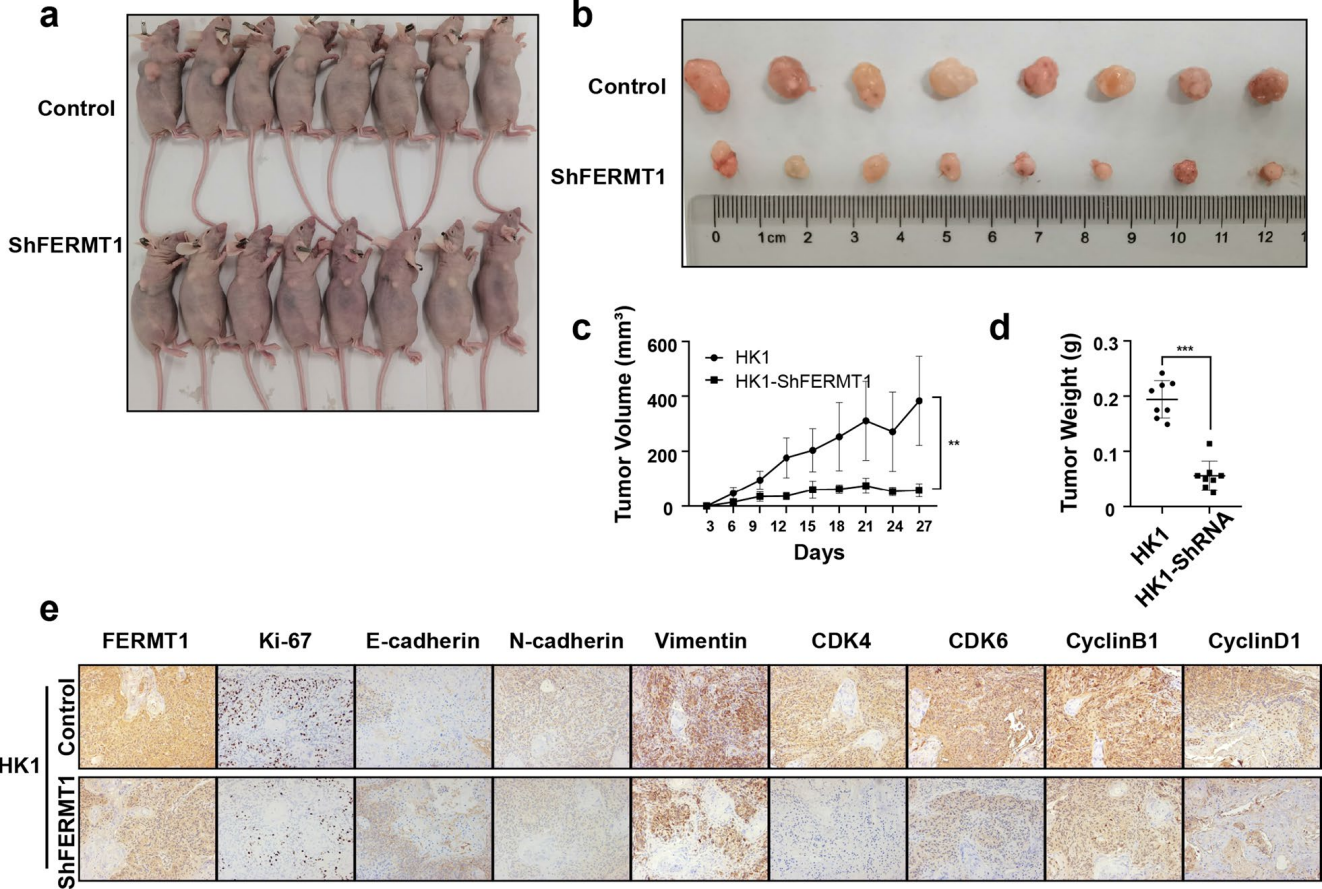
Knockdown of FERMT1 inhibits the growth of xenograft tumors in vivo. **a**, **b** Tumor samples were shown from each group. **c** Tumor volumes from each group were tracked for 27 days. **d** Tumor weight in each group. **e** Representative IHC staining of FERMT1, Ki-67, EMT-related proteins, and cell cycle-related proteins from tumor samples (magnification: 200×); **P < 0.01, ***P < 0.001

Furthermore, we investigated the effect of FERMT1 on the metastasis of NPC cells in vivo. To this end, HK1 cells and stable FERMT1 knockdown cells were inoculated into nude mice, and tumor growth was monitored every 3 days. Through the measurement 27 days after inoculation with NPC cells, we found that the average tumor volume of the HK1-shFERMT1 group was significantly lower than that of the control group (P < 0.01; Fig. 8a–c). At the end of the experiment, we found that the mean tumor weight of the shFERMT1 group was lighter than that of the control group (P < 0.001; Fig. 8d). IHC was applied to the tumor sections of the HK1-shFERMT1 and control groups. As shown in Fig. 8e, the percentage of Ki-67-positive cells in HK1-shFERMT1 xenograft tumors was significantly lower than that in the control group. Additionally, FERMT1 knockdown suppressed EMT in vivo, as evidenced by the upregulation of E-cadherin and the downregulation of N-cadherin and vimentin. In addition, we found that FERMT1 knockdown could inhibit cell cycle checkpoints, including CDK4, CDK6, cyclinB1 and cyclinD1, in vivo.

## Discussion

Nasopharyngeal carcinoma (NPC) progression is a multifactorial complex disease that includes distinct genetic and epigenetic alterations. Distant metastasis remained a major barrier for patient outcome improvement with NPC. In this study, we demonstrated that FERMT1 expression was upregulated in NPC tissues and cell lines. Our study found that FERMT1 promoted NPC proliferation, migration and invasion by regulating EMT and cell cycle progression.

FERMT1, FERMT2, and FERMT3 (also known as Kindlin-1, Kindlin-2, Kindlin-3), three focal adhesion proteins, composed of the 4.1-ezrin-radixin-moesin (FERM) domains, are evolutionarily conserved [22, 23]. Deleting FERMT1 leads to Kindler syndrome characterized by skin abnormalities, which is related to an increased risk of developing squamous cell carcinomas [24, 25]. At present, there are no reports of human FERMT2 functional deletion mutations. Early gene ablation research that used zebra fishes and mice as animal models found that deletion of FERMT2 is fatal to embryos [26, 27]. Deleting FERMT3 function leads to leukocyte adhesion deficiency type III disease, characterized by a propensity to bleed and an impaired immune system [28]. Aberrant expression of kindlins has been investigated in several types of cancer [29]. As previously mentioned, aberrant FERMT1 expression has been reported in various human cancers. FERMT1 is overexpressed in many cancers, including colon cancer, breast cancer, lung cancer, hepatocellular carcinoma, and pancreatic cancer [30]. It seems likely that FERMT1 is a tumor driver in cutaneous epithelial cells. The processes and mechanisms are complex, in which uncontrolled proliferation, invasion, and metastasis are the most important properties of malignant tumors. A previous study by Sin et al. reported that FERMT1 promoted EMT progression and lung cancer metastasis by activating TGF-β signaling [31]. Among these cancers, TGF-β signaling appeared to be an important factor in the carcinogenic effect of FERMT1. Emanuel et al. found that FERMT1 could regulate cutaneous stem cell proliferation by controlling the transcription of Wnt ligands and receptors [32]. In addition, it seemed that immunolabeling of FERMT1 and β-catenin colocalized the intestinal epithelium [33]. In the present study, through GEO database analysis, we demonstrated that FERMT1 expression was higher in NPC tissues than in normal tissues. In NPC tissues, the expression level of FERMT1 was significantly correlated with the clinical stage of NPC patients, suggesting that FERMT1 might be involved in the progression of human NPC metastasis. Additionally, the overall survival time of NPC patients with high FERMT1 expression was shorter. These results indicated that FERMT1 might be a prognostic indicator in patients with NPC.

Furthermore, we investigated the relationship between FERMT1 and NPC cells. Our results indicated that the expression of FERMT1 was markedly higher in NPC cells. Yan et al. showed that overexpression of FERMT1 by a lentiviral vector played an important role in facilitating esophageal cancer cell proliferation and radiation resistance in vitro and promoting tumor growth in vivo [34]. Thus, we hypothesized that FERMT1 depletion could suppress NPC cell proliferation, migration and invasion. We knocked down FERMT1 expression by lentiviral shRNA and subsequently found that FERMT1 expression depletion suppressed NPC cell proliferation and tumor growth in vitro and in vivo. Numerous studies had suggested that EMT progression contributed to the early stage and was pivotal for NPC invasion and metastasis. A study by Liu et al. indicated that FERMT1 promoted EMT in colon cancer cells in vitro and in vivo [5]. In addition, a study demonstrated that FERMT1 promoted EMT in gastric cancer cells by activating the NF-κB signaling pathway via cell and animal experiments. Here, we found that FERMT1 knockdown could remarkably suppress EMT-related molecular biomarkers closely related to NPC progression and metastasis.

We also investigated whether FERMT1 could affect the cell cycle progression of NPC cells. After combining cyclin D1 and cyclin-dependent kinase 4/6 (CDK4/6) to form a complex, retinoblastoma (RB) protein is phosphorylated and releases the E2F transcription factor in the nucleus, which enables downstream genes to initiate transcription, thereby promoting cell cycle progression from G1 to S phase [35–37]. The results showed that knockdown of FERMT1 inhibited cell cycle development from G0/G1 to S phase. At the molecular level, the expression of p-Rb, cyclin B1, CDK4, and CDK6 was suppressed by FERMT1. The induction of cell cycle arrest explained FERMT1-mediated growth suppression in NPC. Hence, through regulating EMT and the cell cycle, FERMT1 played a role in NPC cell growth and metastasis.

To further explore the mechanism by which FERMT1 regulated EMT of NPC cells, we found that the expression of FERMT1 had a positive correlation with NLRP3 in HK1 and CNE2 cells by GSEA analysis. NLRP3 was one of the components of a polyprotein complex called the inflammasome, which was involved in the immune responses against pathogens and self-antigens by amplifying NF-κB and MAPK signaling pathway [38, 39]. The activation of NLRP3 inflammasome was related to the tumor pathogenesis [40, 41]. Shao et al. indicated that NLRP3 knockdown inhibited migration and growth in colorectal cancer cells, and reversed EMT in vitro [42]. Our study was consistent with their results, knockdown FERMT1 inhibited EMT through directly binding to the NLRP3 and inhibited NF-kB signaling pathway. Although the molecular mechanism suggested that FERMT1 could regulate the expression levels of markers related to cell migration, its specific detailed regulatory mechanism remained to be further explored.

## Conclusions

In conclusion, our results suggested that FERMT1 played an important role in the metastasis of NPC. Silencing FERMT1 expression in NPC could reduce tumor proliferation, migration and invasion by reversing EMT and the cell cycle in vitro and in vivo. Knockdown FERMT1 inhibited EMT through directly binding to the NLRP3 and inhibited NF-kB signaling pathway. Therefore, these results indicated that FERMT1 could be a potential biomarker in the treatment of NPC.

## Supplementary Information

**Additional file 1:** **Table S1.** Sequences of primers used for quantitative real-time polymerase chain reaction. **Table S2.** Univariate and multivariate analysis of clinicopathological characteristics of NPC patients’ overall survival (OS)

## Data Availability

The data and materials in the current study are available from the corresponding author on reasonable request.

## Abbreviations

FERMT1: Fermitin family member 1
NPC: Nasopharyngeal carcinoma
EMT: Epithelial–mesenchymal transition
KS: Kindler syndrome
CDK: Cyclin-dependent kinase
IHC: Immunohistochemistry
RIPA: Radioimmunoprecipitation assay
SDS–PAGE: Sodium-dodecyl-sulfate polyacrylamide gel electrophoresis
PVDF: Polyvinylidene fluoride
qRT-PCR: Quantitative real-time polymerase chain reaction
CCK-8: Cell Counting Kit‐8
SPF: Specific pathogen-free
GEO: Gene Expression Omnibus
GSEA: Gene set enrichment analysis
NES: Normalized enrichment score
FDR: False discovery rate
DEGs: Differentially expressed genes
