# Supplementary material for: FERMT1 contributes to the migration and invasion of nasopharyngeal carcinoma through epithelial–mesenchymal transition and cell cycle arrest
Source: Cancer Cell Int. 2022 Feb 10;22:70. doi: 10.1186/s12935-022-02494-1 (PMC8832859; doi:10.1186/s12935-022-02494-1)
Supplement: Supplementary file 1 — Additional file 1: Table S1. Sequences of primers used for quantitative real-time polymerase chain reaction. Table S2. Univariate and multivariate analysis of clinicopathological characteristics of NPC patients’ overall survival (OS) [file 12935_2022_2494_MOESM1_ESM.docx]

Table S1. Sequences of primers used for quantitative real-time polymerase chain reaction

| **Gene** | **Forward or reverse** | **Sequences** |
| --- | --- | --- |
| FERMT1 | Forward | AAGATGGTGAGGTTGCGAGTC |
|  | Reverse | ATGGTGGATGATGCTGGTGTT |
| GAPDH | Forward | CAGGAGGCATTGCTGATGAT |
|  | Reverse | GAAGGCTGGGGCTCATTT |
| E-cadherin | Forward | ATTTTTCCCTCGACACCCGAT |
|  | Reverse | TCCCAGGCGTAGACCAAGA |
| N-cadherin | Forward | TGCGGTACAGTGTAACTGGG |
|  | Reverse | GAAACCGGGCTATCTGCTCG |
| Vimentin | Forward | GACGCCATCAACACCGAGTT |
|  | Reverse | CTTTGTCGTTGGTTAGCTGGT |

Table S2 Univariate and multivariate analysis of clinicopathological characteristics of NPC patients’ overall survival (OS)

|  | Univariate analysis | |  | Multivariate analysis | |
| --- | --- | --- | --- | --- | --- |
|  | HR (95% CI) | P value |  | HR (95% CI) | P value |
| FERMT1 expression | 2.830 (0.886-9.040) | 0.079 |  |  |  |
| Age | 1.449 (0.404-5.195) | 0.569 |  |  |  |
| Gender | 6.688 (0.874-51.21) | 0.067 |  |  |  |
| T classification | 1.657 (0.912-3.009) | 0.097 |  |  |  |
| N classification | 2.992 (1.385-6.463) | 0.005 |  | 2.149 (0.985-4.687) | 0.055 |
| M classification | 4.317 (1.155-16.132) | 0.03 |  | 1.927 (0.486-7.640) | 0.351 |
| clinical stage | 3.704 (1.332-10.303) | 0.012 |  | 2.402 (0.761-7.584) | 0.135 |
| Histologic subtypes | 24.117 (0.016-367.222) | 0.395 |  |  |  |

NPC, nasopharyngeal carcinoma; HR, Hazards ratio; 95% CI, 95% confidence interval
